# Assessing the benefits and safety profile of incorporating poly ADP-ribose polymerase (PARP) inhibitors in the treatment of advanced lung cancer: a thorough systematic review and meta-analysis

**DOI:** 10.3389/fphar.2024.1338442

**Published:** 2024-06-21

**Authors:** Min Tang, Yue Wang, Pulin Li, Rui Han, Ran Wang

**Affiliations:** ^1^ Department of Respiratory and Critical Care Medicine, The First Affiliated Hospital of Anhui Medical University, Hefei, China; ^2^ Department of Infectious Disease, Hefei Second People’s Hospital, Hefei, China

**Keywords:** polyadenosine diphosphoribose polymerase inhibitors, overall survival, progression-free survival, lung cancer, adverse events

## Abstract

**Background:**

Poly (ADP-Ribose) Polymerase (PARP) inhibitors represent a novel class of drugs that hinder DNA repair mechanisms in tumor cells, leading to cell death. This systematic review aims to evaluate the effectiveness, safety, and potential adverse effects of PARP inhibitors (PARPi) in the management of patients with advanced lung cancer.

**Materials and Methods:**

We conducted a comprehensive search for relevant studies in PubMed, Embase, Cochrane, and ClinicalTrials.gov. We extracted primary and secondary outcome measures, including progression-free survival (PFS), overall survival (OS), and adverse events (AEs), from the identified literature for subsequent meta-analysis and systematic review.

**Results:**

This study encompassed twelve randomized controlled trials, involving 3,132 patients with advanced lung cancer. In comparison to non-PARPi treatments, the administration of PARPi significantly extended OS (hazard ratio (HR) = 0.90, 95% CI = 0.83–0.97, *p* = 0.006). However, the difference in PFS did not reach statistical significance.

**Conclusion:**

In summary, therapies incorporating PARPi provide a degree of benefit by extending OS in patients with advanced lung cancer. Nonetheless, further trials are necessary to furnish additional evidence regarding the efficacy and safety of PARPi in the treatment of lung cancer.

**Systematic Review Registration:** https://www.crd.york.ac.uk/PROSPERO/, identifier number: CRD42023424673.

## 1 Background

Cancer is a global health challenge, with lung cancer standing out as the most common malignancy, accounting for 11.6% of all diagnosed cases (Leiter et al., 2023). In 2022, it was estimated that the United States would see approximately 236,740 new cases of lung cancer, leading to around 130,180 fatalities (Siegel et al., 2023). Lung cancer is broadly categorized into two histological types: Small cell lung cancer (SCLC) and Non-small cell lung cancer (NSCLC) (Howlader et al., 2020). NSCLC can be further divided into four distinct subtypes: LUAD, Lung squamous cell carcinoma (LUSC), Large-Cell Carcinoma, and Bronchial Carcinoid Tumor. LUAD is the most prevalent subtype of NSCLC and the most common primary lung tumor (Dragoj et al., 2019; Han et al., 2023). While surgical intervention is recommended for patients with stage I–II non-small-cell lung cancer (Vansteenkiste et al., 2014), early detection is often lacking, resulting in diagnoses at later stages characterized by local tumor invasion or distant metastasis, making surgical treatment impractical. Currently, platinum-based chemotherapy regimens, such as carboplatin and paclitaxel combinations, remain the standard of care for NSCLC patients (Ohe et al., 2007; Planchard et al., 2018). Recent advancements in lung cancer diagnosis and treatment, particularly the increased detection of cancer driver genes through genomic analysis, have led to the emergence of targeted therapies as primary treatments for lung cancer patients (Zhang et al., 2021). Drugs targeting EGFR and ALK-positive mutations have seen multiple generations of development and are widely used as first-line treatments, significantly improving the prognosis of patients with driver gene-positive NSCLC (Miller et al., 2022). SCLC, which accounts for 15%–20% of all primary lung cancers, is characterized by its aggressive nature, often linked to smoking. It exhibits a rapid doubling time, a high growth fraction, and early development of widespread metastases (AKP et al., 2021; Wong and Iams, 2021). Consequently, chemotherapy, often combined with chest radiotherapy, remains the cornerstone of treatment for SCLC patients (Horn et al., 2016). Immunotherapy has also emerged as a promising avenue for lung cancer treatment in recent years (Somasundaram and Burns, 2017; Li et al., 2018; Zhang et al., 2020).

DNA damage, a common occurrence throughout the cell life cycle, arises from single-strand breaks (SSB) or double-strand breaks (DSB). If left unrepaired, this damage can lead to genomic instability and ultimately cell death (Lau et al., 2022). Humans have developed several key DNA repair pathways to combat this damage: base excision repair (BER), nucleotide excision repair (NER), mismatch repair (MMR), homologous recombination (HR), non-homologous end joining (NHEJ), translesion synthesis (TLS), and interstrand crosslink (ICL) repair. BER is primarily responsible for repairing SSBs, whereas HR and NHEJ are the predominant mechanisms for repairing DSBs (Dziadkowiec et al., 2016; Chatterjee and Walker, 2017). Poly (ADP-ribose) polymerase (PARP), a key sensor of DNA damage, disrupts DNA repair mechanisms within tumor cells, playing a crucial role in BER and SSB repair (Brown et al., 2017). In simple terms, they induce genetic-level cell death, aiming to eradicate cancer cells. Genomic instability is caused by high levels of DNA damage due to oxidative or replication stress, defects in DNA repair pathways, and/or dysfunctional monitoring mechanisms that fail to trigger cellular senescence or apoptosis (Tubbs and Nussenzweig, 2017). PARP inhibitors exploit a synthetic lethal strategy, enhancing the effects of inherited DNA repair defects with drug-induced impairments in compensatory pathways (Lord and Ashworth, 2017).

Lung tissues are highly exposed to external DNA-damaging agents, such as those found in smoking and air pollution, contributing to the high mutation loads often observed in lung cancers (La Fleur et al., 2019). Homologous repair genes have been implicated in the survival of lung cancer patients post-treatment, with mixed clinical outcomes in cases involving BRCA1 mutations. Research by Margeli, Taron, and their colleagues has demonstrated that lower BRCA1 expression predicts improved outcomes in lung cancer patients (Taron et al., 2004; Margeli et al., 2010). Approximately 14% of NSCLC patients carry the BRCA1/2 mutation, compared to about 12% in SCLC (26). Inactivation of TP53 and RB1 is prevalent in SCLC genomes, making tumor cells more reliant on DNA damage repair mechanisms. Lung cancer cells depleted of these genes are highly sensitive to PARPi, particularly olaparib, and exhibit apoptosis when exposed to the drug (Ji et al., 2020). Notably, nuclear enzymes PARP-1 and PARP-2 play key roles in recognizing and facilitating DNA damage repair (Amé et al., 2004; Lord and Ashworth, 2012). Research has revealed high PARP-1 expression in SCLC, indicating its sensitivity to platinum-based chemotherapy, aligning with the therapeutic mechanism of PARPi (Byers et al., 2012).

Despite the encouraging treatment results, it is inevitable that many patients will develop resistance to PARPi (Kaur et al., 2022; Cai et al., 2023). One of the most prevalent mechanisms behind PARPi acquired resistance is secondary mutations that replace homologous recombination repair (HRR) function (Barber et al., 2013). In addition, protection of DNA replication forks, expression of different variants of BRCA-1, and demethylation of BRCA-1 and RAD 51C promoter regions also play key roles in resistance to PARPi (Drost et al., 2016; Ray Chaudhuri et al., 2016; Ter Brugge et al., 2016). Therefore, how PARPi’s involvement in processes unrelated to DNA repair affects PARPi’s anti-cancer effects will help in the development of drugs that overcome PARPi resistance and increase PARPi sensitivity.

PARPi have gained prominence as potential therapeutics for various diseases, including cancer. Olaparib received approval for standalone use from the European Medicines Agency (EMA) in the European Union and the United States Food and Drug Administration (FDA) in 2014 (Kim et al., 2015). Subsequently, other drugs like rucaparib (Du et al., 2016), niraparib (Moore et al., 2019), talazoparib (Coleman et al., 2017) and veliparib (Han et al., 2022) have also been introduced. PARPi have made significant strides in oncology, obtaining FDA approval for the treatment of breast, ovarian, and prostate cancers. However, their efficacy in thoracic malignancies, such as NSCLC and SCLC, has not yielded similar results.

Therefore, this study incorporates findings from twelve trials to comprehensively evaluate the effectiveness and safety of integrating poly ADP-ribose polymerase (PARP) inhibitors into the therapy for advanced lung cancer.

## 2 Methods

### 2.1 Protocol and guidance

This study adhered to the guidelines outlined by the Preferred Reporting Items for Systematic Reviews and Meta-Analyses (PRISMA). The research protocol for this systematic review was registered with PROSPERO, an international registry dedicated to prospective systematic reviews (Registration No: CRD42023424673).

### 2.2 Data retrieval and search strategy

A comprehensive search was conducted by two independent investigators across the following databases: PubMed, Embase, Cochrane, and ClinicalTrials.gov. The search spanned from the inception of these databases up to 7 May 2023. The search terms employed included “PARP inhibitor,” “poly ADP-ribose polymerase inhibitor,” “lung cancer,” “lung neoplasm,” “olaparib,” “rucaparib,” “talazoparib,” “veliparib,” and “niraparib.” No restrictions were imposed concerning countries, authors, or language. All search queries incorporated both Medical Subject Headings (MeSH) and free-text keywords. The full search string can be found in the Supplementary Materials for reference. To ensure the comprehensiveness of our search, we also cross-referenced the proceedings of the American Society of Clinical Oncology (ASCO) and the European Society for Medical Oncology (ESMO) conferences to confirm the inclusion of all eligible articles. In cases of duplicate publications, only the most comprehensive or the most recent report of a clinical trial was considered for inclusion in the meta-analysis.

### 2.3 Study selection

We followed specific criteria for the selection of clinical trials for inclusion in this study:

### 2.4 Inclusion criteria

• The trial was reported in English and constituted a completed clinical randomized trial evaluating the efficacy of PARPi.
• The trial fell into either Phase II or Phase III randomized controlled trials (RCTs) involving the use of PARPi.
• The included study reported data on primary or secondary outcome measures, specifically OS or PFS.

### 2.5 Exclusion criteria

• Trials were excluded if the publication was a case report, review, meta-analysis, retrospective study, or presented data from animal or *in vitro* testing.
• Articles presented solely as abstracts without the availability of the full original text were also excluded from consideration.
• These criteria ensured a rigorous selection process to maintain the quality and relevance of the studies included in our analysis.

### 2.6 Data abstraction

Data abstraction was carried out independently by two investigators, and any discrepancies were resolved through consensus. For each included study, the following information was extracted:

• First author’s name
• Year of publication
• Trial phase
• Registration number
• Type of PARP inhibitor used
• Description of treatment arms and control arms
• Number of patients in each treatment arm
• mPFS
• mOS

The assessment of severe AEs, including all grades and those categorized as grade ≥3, was conducted based on the safety profile reported in each trial. AEs data were recorded in accordance with either version 3.0 or 4.0 of the Common Terminology Criteria for Adverse Events (CTCAE), which can be accessed at http://ctep.cancer.gov.

This systematic and comprehensive approach ensured the accurate extraction of relevant data from each included study, contributing to the robustness of our analysis.

### 2.7 Bias assessment

The risk of bias in the studies included in this review was assessed following the Cochrane Intervention Systems Review Manual. We utilized six criteria for this assessment:

• Random Sequence Generation: We evaluated the method used for generating random sequences and categorized it as low, unclear, or high risk of bias.
• Allocation Concealment: The degree of allocation concealment was examined and categorized as low, unclear, or high risk of bias.
• Blindness of Participants and Researchers: We assessed the level of blinding of both participants and researchers involved in the studies and categorized it as low, unclear, or high risk of bias.
• Blindness of Outcome Assessment: We evaluated the extent to which outcome assessment was blinded and categorized it as low, unclear, or high risk of bias.
• Incomplete Outcome Data: An assessment was made regarding the handling of incomplete outcome data, and we categorized it as low, unclear, or high risk of bias.
• Selective Reporting: We determined the risk of selective reporting bias for each study. Additionally, funnel plots were used to assess the potential presence of publication bias in the included studies.

### 2.8 Statistical analysis

Hazard ratios (HRs) with corresponding 95% confidence intervals (CIs) were used to assess both OS and PFS among patients with advanced or metastatic lung cancer who received treatment with PARPi. For the evaluation of AEs, relative risks (RRs) with 95% CIs were employed.

Two primary statistical models were utilized in this meta-analysis: the fixed-effect model, weighted by inverse variance, and the random-effect model. The choice between these models was determined based on heterogeneity assessments. Heterogeneity among the included studies was evaluated using the χ^2^ test and the I^2^ statistic. When the I^2^ statistic exceeded 50% or when the *p*-value was less than 0.1, heterogeneity was considered statistically significant, leading to the adoption of the random-effect model. Conversely, in the absence of substantial heterogeneity, the fixed-effect model was applied.

We conducted sensitivity analysis of PFS and OS outcomes by systematically omitting individual studies from the meta-analysis to test the robustness and stability of the results. Statistical significance was determined based on a threshold of a *p*-value less than 0.05. All extracted data were meticulously recorded in an Excel spreadsheet and subjected to statistical analysis using Stata 16.0, a software package developed by the U.S. Computer Resource Center.

## 3 Results

### 3.1 Literature search and eligible studies

**FIGURE 1:**
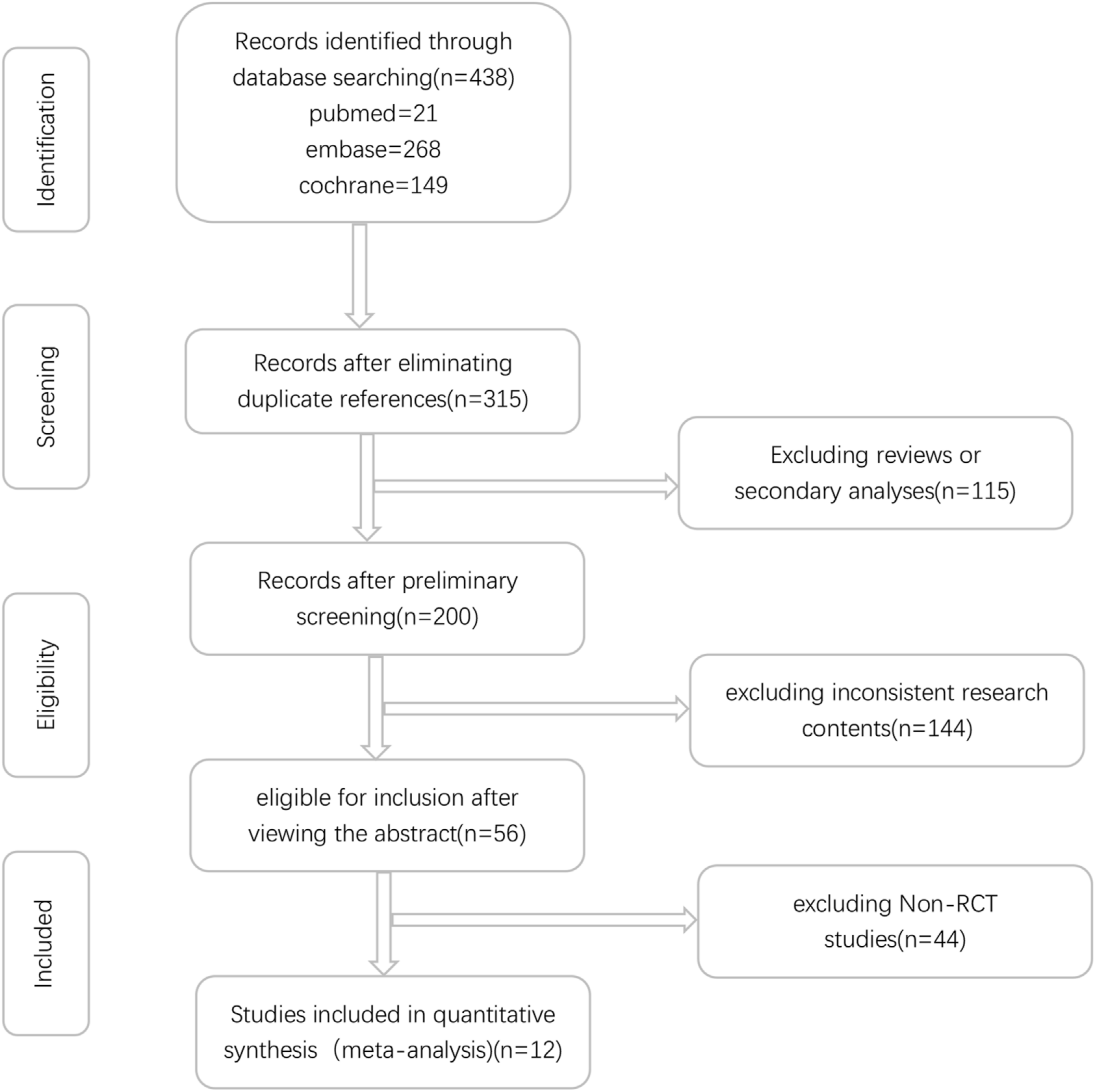
Flowchart of literature screening.

Initially, a total of 438 relevant studies were identified through electronic searches. Among these, 123 studies were excluded as duplicates. Subsequently, an evaluation of the titles and abstracts led to the exclusion of an additional 144 and 115 studies, respectively. These exclusions were based on non-compliance with the inclusion criteria or because the studies fell under the review category. A detailed depiction of the search and selection process can be found in Figure 1. It is worth noting that Iniparib, initially considered a PARP inhibitor but later revealed to act via non-selective protein modification through cysteine adducts, was excluded from consideration (Novello et al., 2014). Additionally, the Phase II randomized PIPSeN trial, led by S. Postel-Vinay et al., was prematurely terminated and lacked sufficient statistical data for inclusion, leading to its exclusion (Postel-Vinay et al., 2021). After a thorough examination of the full texts of the remaining 56 studies, a total of 12 randomized controlled trials (RCTs) (Chabot et al., 2017; Ramalingam et al., 2017; Pietanza et al., 2018; Owonikoko et al., 2019; Garcia-Campelo et al., 2020; Ai et al., 2021; Argiris et al., 2021; Byers et al., 2021; Ramalingam et al., 2021; Fennell et al., 2022; Govindan et al., 2022; Woll et al., 2022), involving 3,132 patients, were ultimately included in this meta-analysis (Table 1).

**TABLE 1:** Summary of included studies.

Study	Year	Classification	Intervention group	Control group	Registration number	Phase	Population	The primary endpoint
Ramalingam et al. (2017)	2017	NSCLC	Veliparib + PC	Placebo + PC	NCT01560104	II	158	PFS
Chabot et al. (2017)	2017	NSCLC	Veliparib + WBRT	Placebo + WBRT	NA	II	307	OS
Garcia-Campelo et al. (2020)	2020	NSCLC	Olaparib + Gefitinib	Gefitinib	NCT01513174	II	182	PFS
Argiris et al. (2021)	2021	NSCLC	Veliparib + CRT	Placebo + CRT	NCT01386385	II	32	PFS
Ramalingam et al. (2021)	2021	NSCLC	Veliparib + PC	Placebo + PC	NCT02106546	III	970	OS
Govindan et al. (2022)	2022	NSCLC	Veliparib + PC	PC/MC	NCT02264990	III	595	OS
Fennell et al. (2022)	2022	NSCLC	Olaparib	Placebo	NCT01788332	II	70	PFS
Pietanza et al. (2018)	2018	SCLC	Veliparib + Temozolomide	Placebo + Temozolomide	NCT01638546	II	104	PFS
Owonikoko et al. (2019)	2019	SCLC	Veliparib + EC	Placebo + EC	NCT01642251	II	128	PFS
Ai et al. (2021)	2021	SCLC	Niraparib	Placebo	NCT03516084	III	185	PFS + OS
Byers et al. (2021)	2021	SCLC	Veliparib + EC	Placebo	NCT02289690	II	181	PFS
Woll et al. (2022)	2022	SCLC	Olaparib	Placebo	NA	II	220	PFS

WBRT, Whole-brain radiation therapy; C, Carboplatin/Cisplatin; P, paclitaxel; E, etoposide; M, pemetrexed; OS, overall Survival; PFS, progression free survival; NA, not available.

### 3.2 Characteristics of included trials and patients

The baseline characteristics of the patient populations included in each study are presented in Table 2. Among the included studies, ten were classified as Phase II trials, and two were designated as Phase III trials. These studies encompassed both SCLC and NSCLC. Specifically, five studies pertained to SCLC, while the remaining seven focused on NSCLC.

**TABLE 2:** Baseline characteristics of the study populations.

Study	Year	Classification	Group	Population	Age (range), y	Male, n (%)	White, n (%)	Smoking, n (%)	EGOC 0–1, n (%)
Ramalingam et al. (2017)	2017	NSCLC	Veliparib + PC	105	63 (33–84)	75 (71)	102 (97)	45 (85)	53 (100)
			Placebo + PC	53	62 (46–79)	32 (60)	52 (98)	92 (88)	105 (100)
Chabot et al. (2017)	2017	NSCLC	Veliparib 50 mg + WBRT	103	60 (33–83)	61 (59)	85 (83)	85 (82)	NA
			Veliparib 200 mg + WBRT	102	62 (39–81)	66 (65)	66 (65)	78 (77)	NA
			Placebo + WBRT	102	60 (41–86)	56 (55)	79 (78)	77 (76)	NA
Garcia-Campelo et al. (2020)	2020	NSCLC	Olaparib + Gefitinib	91	65 (39–85)	25 (27)	NA	31 (34)	84 (92)
			Gefitinib	91	68 (36–85)	34 (37)	NA	36 (40)	83 (91)
Argiris et al. (2021)	2021	NSCLC	Veliparib + CRT	18	65 (47–79)	7 (39)	13 (72)	9 (50)	18 (100)
			Placebo + CRT	13	65 (57–76)	7 (54)	12 (92)	6 (46)	13 (100)
Ramalingam et al. (2021)	2021	NSCLC	Veliparib + PC	485	64 (36–83)	411 (85)	471 (97)	457 (94)	486 (100)
			Placebo + PC	484	64 (33–84)	384 (79)	477 (99)	457 (94)	484 (100)
Govindan et al. (2022)	2022	NSCLC	Veliparib + PC	298	63 (27–81)	206 (69)	229 (77)	298 (100)	298 (100)
			PC/MC	297	64 (34–85)	207 (70)	233 (78)	297 (100)	297 (100)
Fennell et al. (2022)	2022	NSCLC	Olaparib	32	65 (61–72)	16 (50)	NA	29 (91)	32 (100)
			Placebo	38	63 (59–70)	24 (63)	NA	35 (92)	38 (100)
Pietanza et al. (2018)	2018	SCLC	Veliparib + Temozolomide	55	63 (31–80)	24 (44)	NA	49 (89)	55 (100)
			Placebo + Temozolomide	49	62 (35–84)	26 (53)	NA	44 (90)	49 (100)
Owonikoko et al. (2019)	2019	SCLC	Veliparib + EC	64	66 (59–72)	34 (53)	61 (95)	NA	64 (100)
			Placebo + EC	64	64 (59–70)	32 (50)	57 (89)	NA	64 (100)
Ai et al. (2021)	2021	SCLC	Niraparib	125	61.0 ± 8.86	101 (81)	NA	NA	NA
			Placebo	60	61.5 ± 6.56	49 (82)	NA	NA	NA
Byers et al. (2021)	2021	SCLC	Veliparib + EC + Veliparib	61	62 (39–77)	40 (66)	55 (90)	60 (98)	60 (98)
			Veliparib + EC + Placebo	59	64 (46–86)	38 (64)	51 (86)	55 (95)	58 (98)
			Placebo	61	63 (37–87)	38 (62)	52 (87)	58 (95)	60 (98)
Woll et al. (2022)	2022	SCLC	Olaparib BID	73	66 (43–89)	36 (49)	NA	NA	68 (93)
			Olaparib TID	73	63 (42–82)	31 (42)	NA	NA	69 (95)
			Placebo	74	64 (43–86)	34 (46)	NA	NA	66 (89)

WBRT, Whole-brain radiation therapy; C, Carboplatin/Cisplatin; P, paclitaxel; E, etoposide; M, pemetrexed; ECOG, eastern cooperative oncology group; BID, twice a day; NA, not available.

Treatment modalities varied among the studies. Three studies employed PARPi as monotherapy, six utilized PARPi in combination with chemotherapy, one integrated PARPi with radiotherapy, another used PARPi in combination with chemo-radiotherapy, and one study incorporated PARPi alongside targeted therapy.

These diverse approaches allowed for a comprehensive assessment of the effectiveness and safety of PARPi in the treatment of advanced lung cancer across different patient populations and treatment regimens.

### 3.3 Efficacy

#### 3.3.1 Overall survival

Among the included studies, ten provided data on OS. However, two studies presented unique challenges in the effect size analysis of OS:

• Pietanza et al. (2018) study lacked the 95% confidence interval for OS.
• Argiris et al. (2021) reported an 80% confidence interval for OS, rendering it ineligible for inclusion in the analysis.

**FIGURE 2:**
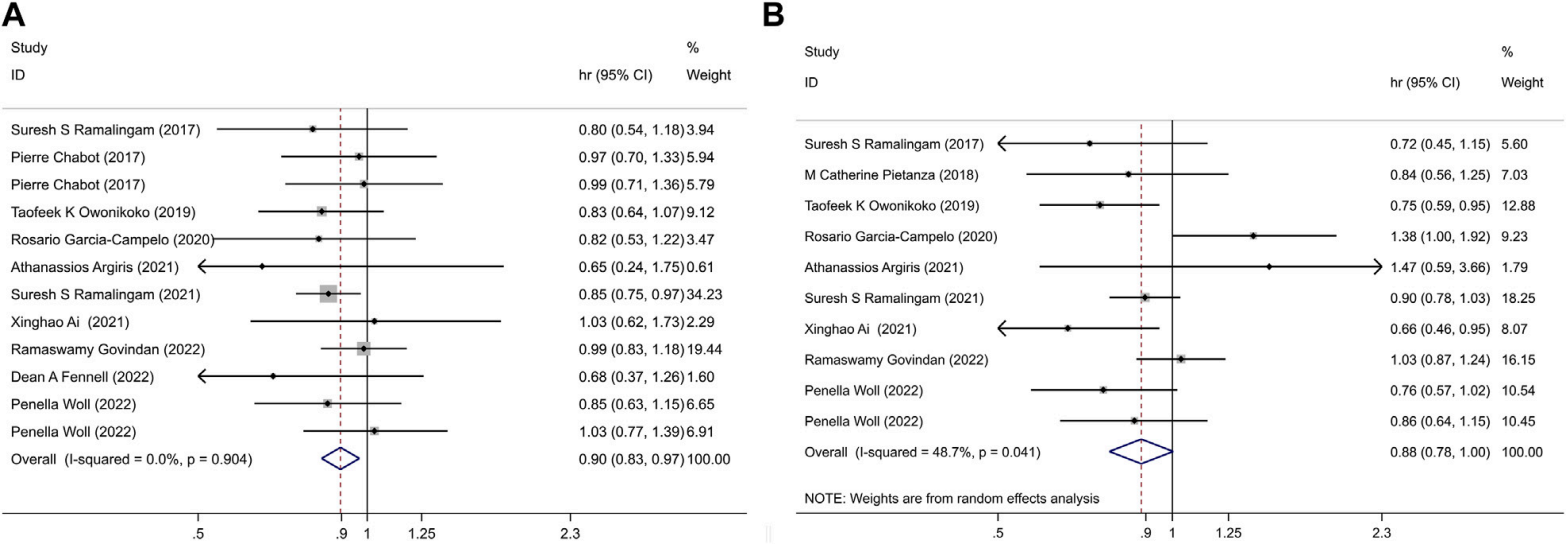
**(A)** PARPi-containing regimen vs. non-PARPi regimen, in LC: OS; **(B)** PARPi-containing regimen vs. non-PARi regimen, in LC: PFS.

Given the absence of significant heterogeneity among the studies (I^2^ = 0, *p* = 0.904), we employed the fixed-effect model for calculations. The results revealed a notable difference in the impact of PARPi on OS between the two groups (HR = 0.90, 95% CI = 0.83–0.97, *p* = 0.006) (Figure 2).

This analysis demonstrated that the incorporation of PARPi into lung cancer treatment was associated with improved OS when compared to non-PARP inhibitor treatments.

**FIGURE 3:**
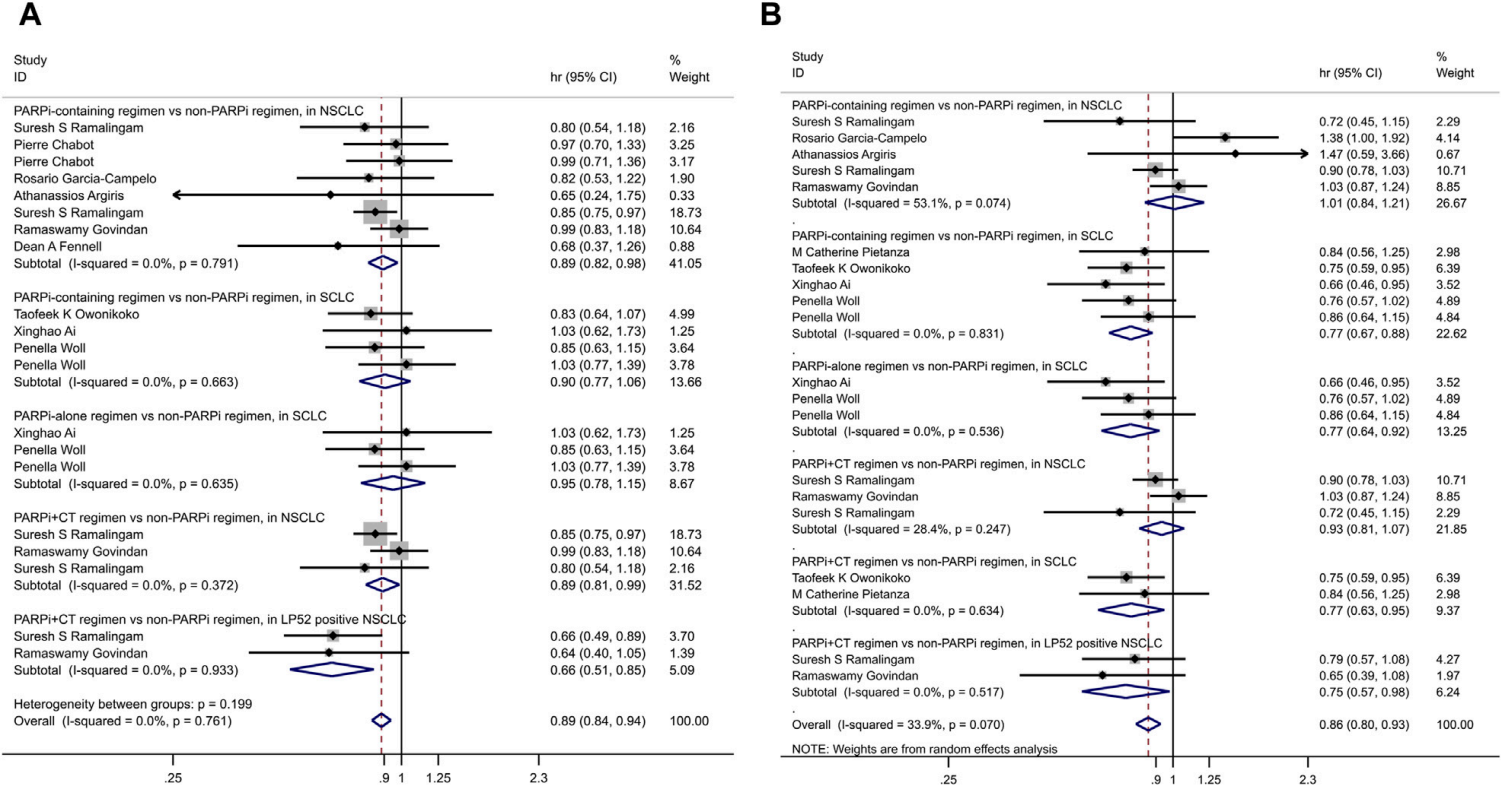
The forest maps for each subgroup analysis. **(A)** OS; **(B)** PFS.

Subgroup analyses were conducted based on treatment modalities, revealing the following:

• In patients with NSCLC, treatment regimens containing PARPi extended OS (HR = 0.89, 95% CI = 0.82–0.98, p = 0.014) compared to regimens without PARPi.
• However, no significant survival benefit was observed in patients with SCLC (HR = 0.90, 95% CI = 0.77–1.06, p = 0.205).
• Among patients with NSCLC, when PARPi were combined with chemotherapy, OS improved (HR = 0.89, 95% CI = 0.81–0.99, p = 0.028) compared to non-PARP inhibitor regimens.
• In patients treated with veliparib for NSCLC, this improvement was particularly pronounced in the LP52-positive population (HR = 0.66, 95% CI = 0.51–0.85, p = 0.001) (Figure 3).
• These subgroup analyses highlight the differential impact of PARP inhibitor-containing regimens on OS based on the type of lung cancer (NSCLC vs. SCLC) and the specific treatment modalities employed. Notably, the combination of PARPi with chemotherapy showed a substantial survival advantage in certain patient populations with NSCLC.

#### 3.3.2 Progression free survival

Out of the included studies, a total of nine reported data on PFS. However, similar to the analysis of OS, several studies presented challenges in the analysis. Specifically:

• Chabot et al. (2017) study lacked the 95% confidence interval data for PFS.
• Fennell et al. (2022) and Byers et al. (2021) reported 80% confidence intervals for PFS, rendering them ineligible for inclusion in the analysis.

Given the presence of significant heterogeneity between the trials (I^2^ = 48.7%, *p* = 0.041), we utilized a random-effect model for the meta-analysis. The pooled estimate revealed that, overall, there were no statistically significant differences in PFS between the experimental and control groups (HR = 0.88, 95% CI = 0.78–1.00, *p* = 0.057) (Figure 2). This suggests that the addition of PARPi to the lung cancer treatment regimen did not lead to an extension of PFS compared to non-PARP inhibitor treatments.

Subgroup analyses were conducted based on the type of lung cancer and treatment modalities, yielding the following findings:

• In patients with SCLC, regimens containing PARPi extended PFS(HR = 0.77, 95% CI = 0.67–0.88, p ≤ 0.001) compared to control groups. This benefit was observed in patients with SCLC when PARPi were used either as monotherapy (HR = 0.77, 95% CI = 0.64–0.92, p = 0.004) or in combination with chemotherapy regimens (HR = 0.77, 95% CI = 0.63–0.95, p = 0.013).
• However, in patients with LP52-positive NSCLC, the combination of veliparib with chemotherapy was associated with prolonged PFS (HR = 0.75, 95% CI = 0.57–0.98, p = 0.035) (Figure 3).
• These subgroup analyses provide insights into the differential impact of PARP inhibitor-containing regimens on PFS based on the type of lung cancer and specific treatment approaches. Notably, SCLC patients seemed to benefit from PARPi, while LP52-positive NSCLC patients saw improved outcomes when veliparib were combined with chemotherapy.

### 3.4 Assessment of methodological bias

**FIGURE 4:**
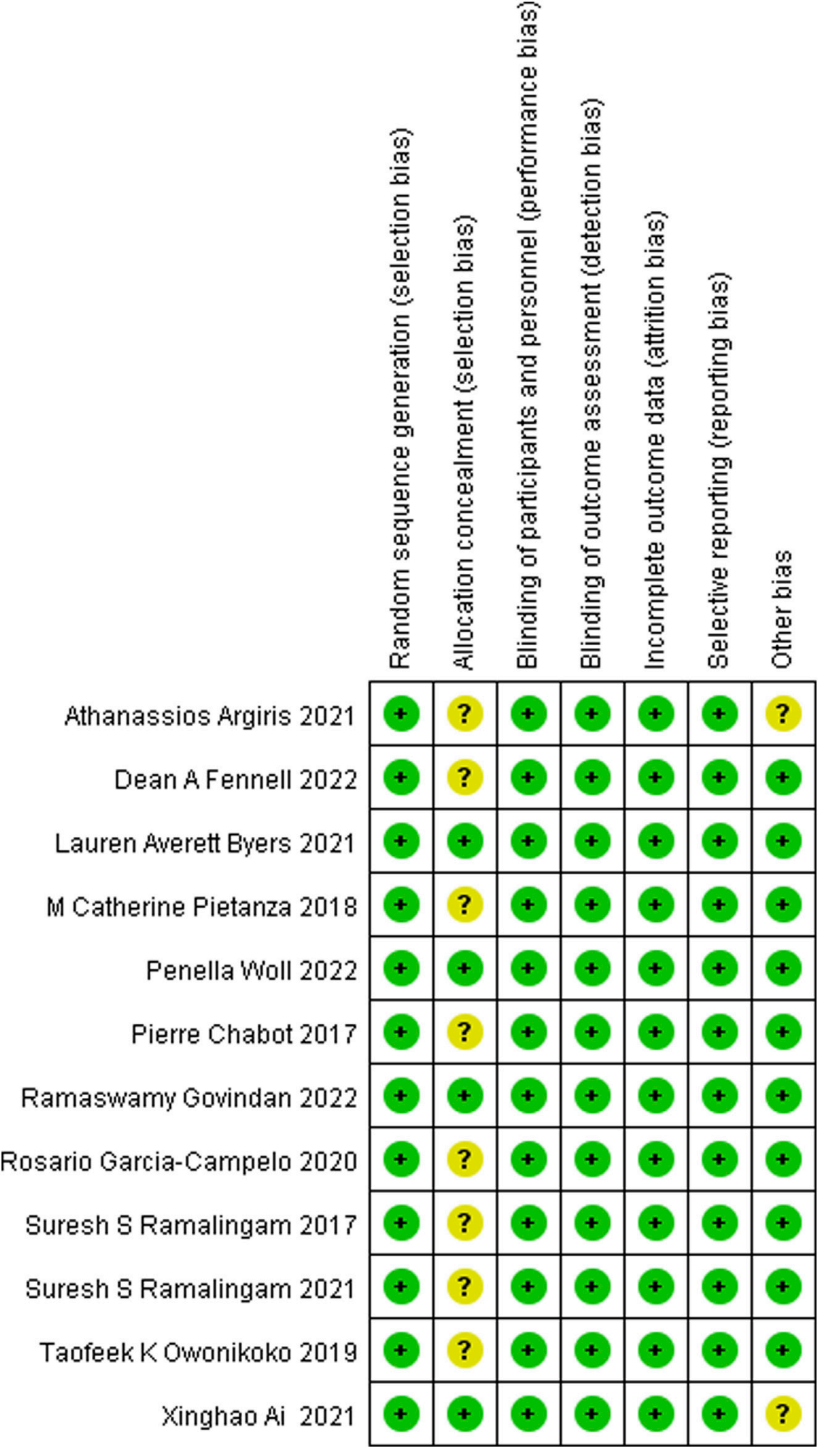
Risk of bias graph: review of authors’ judgements about each risk of bias item presented as percentages across all included studies. Note: each colour represents a different level of bias: red for high risk, green for low risk, and yellow for unclear risk of bias.

The visual presentation of the risk of bias in the studies included in this meta-analysis can be found in Figure 4. It is important to note the following key findings:

• All of the included studies demonstrated appropriate randomization procedures.
• Comprehensive descriptions of allocation concealment were provided in each study.
• Adequate blinding protocols for outcome assessment were reported in all included studies.
• Regarding incomplete outcome data and selective reporting, all studies were assessed to have a low risk of bias in these areas.

These findings collectively indicate that the studies included in this meta-analysis adhered to rigorous methodological standards, contributing to the robustness and reliability of the results obtained.

### 3.5 Sensitivity analysis and publication bias

**FIGURE 5:**
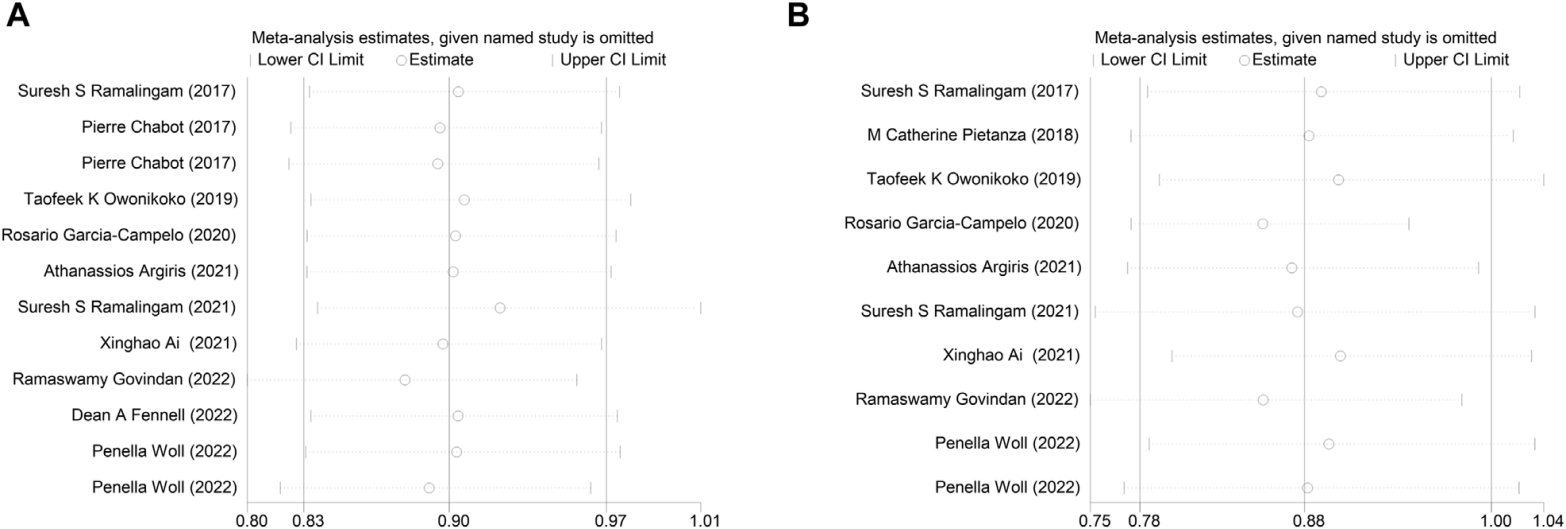
Sensitivity analysis of the **(A)** overall survival (OS), **(B)** progression-free survival (PFS).

Sensitivity analysis was conducted by systematically omitting one study at a time, and the results remained consistent with the overall findings for both OS and PFS (Figure 5). This robustness in the results indicates that the conclusions drawn from the meta-analysis are stable and not reliant on any single study.

**FIGURE 6:**
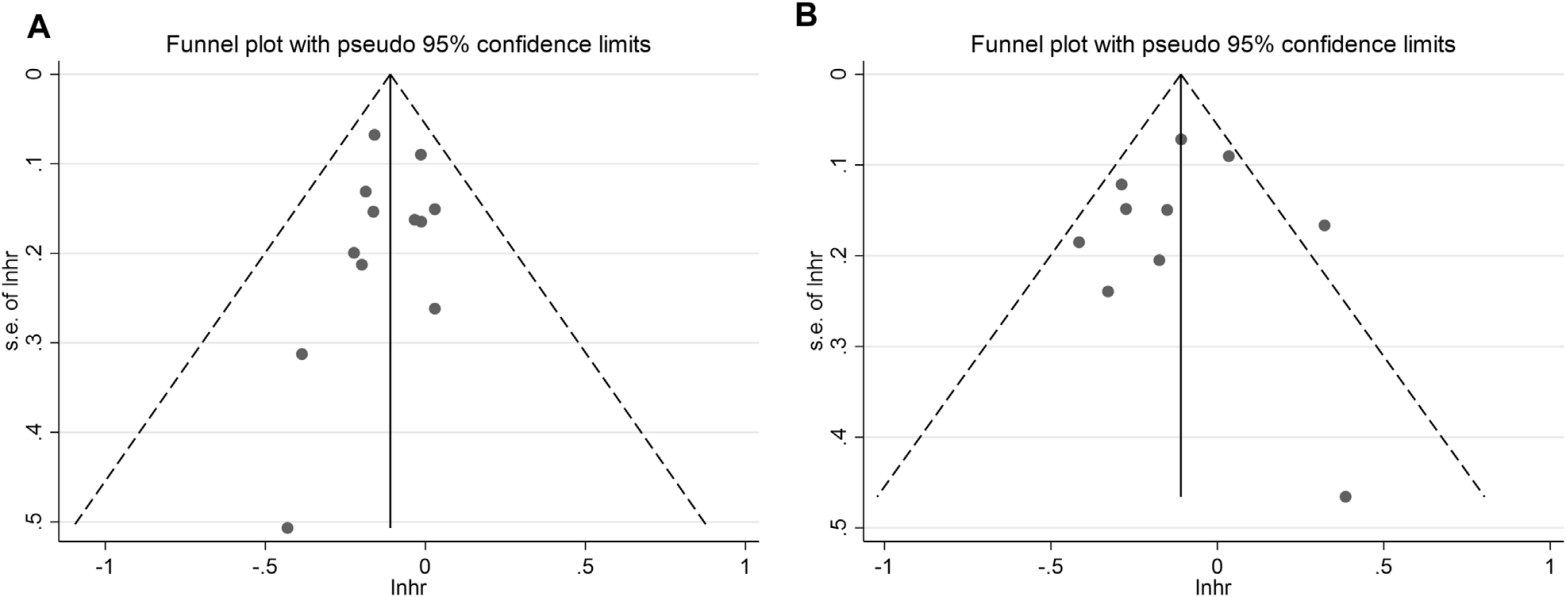
Funnel plot for the **(A)** overall survival (OS), **(B)** progression-free survival (PFS).

To assess the potential for publication bias, the funnel plot test was employed. The analysis revealed low publication bias for both overall OS and PFS (Figure 6). This suggests that the available studies were well-distributed, and the impact of publication bias on the results is likely minimal.

### 3.6 Pooled analysis of adverse events

The pooled analysis, involving a sample size of 3,132 patients, revealed that there was little to no significant difference in the rates of AEs of any grade (RR 1.01, 95% CI 0.99–1.02, *p* = 0.365) and grade 3 or higher AEs (RR 1.03, 95% CI 0.96–1.1, *p* = 0.378).

However, when focusing on serious AEs (grade ≥3), a distinct pattern emerged in patients treated with PARPi. Among the most common hematological AEs reported across all twelve studies were:

• Neutropenia (RR 1.42, 95% CI 1.12–1.81; P = 0.004)
• Anemia (RR 2.24, 95% CI 1.25–3.98; P = 0.006)
• Leukopenia (RR 1.93, 95% CI 1.34–2.76; P < 0.001)

Among the non-hematological AEs, the most common were:

• Nausea (RR 1.58, 95% CI 0.75–3.35; P = 0.229)
• Fatigue (RR 1.21, 95% CI 0.81–1.82; P = 0.349)
• Arthralgia (RR 3.33, 95% CI 0.38–29.22; P = 0.277)
• Decreased appetite (RR 4.08, 95% CI 0.46–35.85; P = 0.205)

**FIGURE 7:**
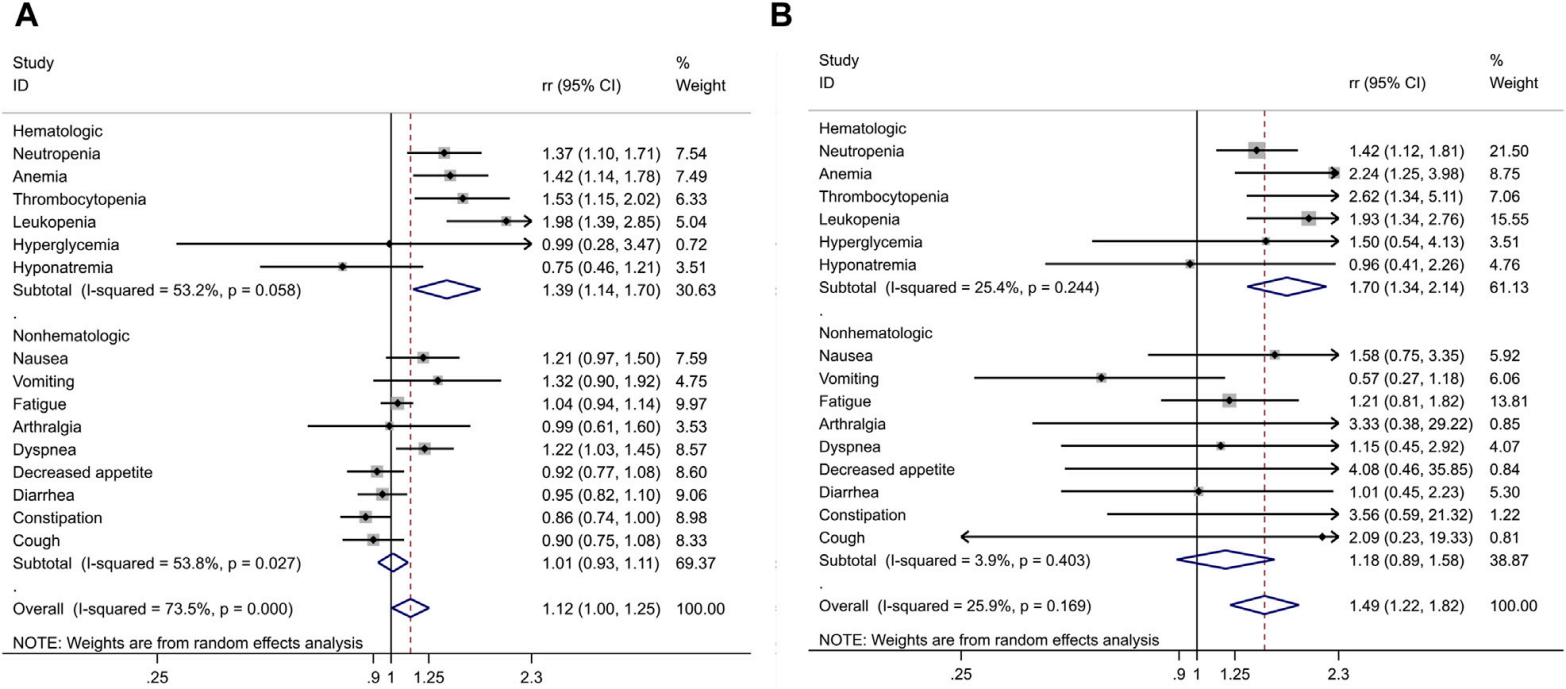
Combined risk ratio (RR) and 95% confidence interval of adverse events included in twelve trials. **(A)** Any grade AE; **(B)** Any grade≥3 AE.

These findings are summarized in Table 3 and Figure 7.

**TABLE 3:** Subgroup analyses of AEs based on treatment.

AE	Group	No. of RCTs	Tests of association			Tests of heterogeneit		
			RR	95%CI	*p*-value	Model	I2	*p*-value
Hematologic								
Neutropenia	Any grade AE	8	1.37	1.1, 1.71	0.006	R	54.2	0.002
	Any grade≥3 AE	7	1.42	1.12, 1.81	0.004	R	43.4	0.079
Anemia	Any grade AE	9	1.42	1.14, 1.78	0.002	R	79.6	<0.001
	Any grade≥3 AE	10	2.24	1.25, 3.98	0.006	R	63.4	0.001
Thrombocytopenia	Any grade AE	8	1.53	1.15, 2.02	0.003	R	59.3	0.008
	Any grade≥3 AE	8	2.62	1.34, 5.11	0.005	R	61.9	0.003
Leukopenia	Any grade AE	6	1.98	1.39, 2.85	<0.001	R	45.4	0.077
	Any grade≥3 AE	8	1.93	1.34, 2.76	<0.001	F	0	0.697
Hyperglycemia	Any grade AE	2	0.99	0.28, 3.47	0.991	R	63.8	0.063
	Any grade≥3 AE	4	1.5	0.54, 4.13	0.432	F	9.3	0.353
Hyponatremia	Any grade AE	2	0.75	0.46, 1.21	0.238	F	0	0.812
	Any grade≥3 AE	6	0.96	0.41, 2.26	0.923	R	58	0.02
Nonhematologic								
Nausea	Any grade AE	10	1.21	0.97, 1.5	0.091	R	77.3	<0.001
	Any grade≥3 AE	6	1.58	0.75, 3.35	0.229	F	0	0.478
Vomiting	Any grade AE	8	1.32	0.9, 1.92	0.153	R	74.8	<0.001
	Any grade≥3 AE	7	0.57	0.27, 1.18	0.128	F	0	0.806
Fatigue	Any grade AE	10	1.04	0.94, 1.14	0.455	F	0	0.582
	Any grade≥3 AE	9	1.21	0.81, 1.82	0.349	F	10.6	0.345
Arthralgia	Any grade AE	5	0.99	0.61, 1.60	0.972	R	63.8	0.011
	Any grade≥3 AE	3	3.33	0.38, 29.22	0.277	F	0	0.956
Dyspnea	Any grade AE	7	1.22	1.03, 1.45	0.018	F	0	0.605
	Any grade≥3 AE	2	1.15	0.45, 2.92	0.777	F	0	0.47
Decreased appetite	Any grade AE	6	0.92	0.77, 1.08	0.306	F	4.97	0.394
	Any grade≥3 AE	2	4.08	0.46, 35.85	0.205	F	0	0.807
Diarrhea	Any grade AE	8	0.95	0.82, 1.10	0.518	F	0	0.702
	Any grade≥3 AE	4	1.01	0.45, 2.23	0.988	F	0	0.67
Constipation	Any grade AE	8	0.86	0.74, 1.00	0.045	F	0	0.959
	Any grade≥3 AE	3	3.56	0.59, 21.32	0.165	F	0	0.957
Cough	Any grade AE	6	0.9	0.75, 1.08	0.267	F	0	0.433
	Any grade≥3 AE	3	2.09	0.23, 19.33	0.515	F	0	0.758

R, random-effect model; F, fixed-effect model; RR, risk ratio; CI, confidence intervals; AE, adverse reaction; RCTs, randomized controlled trials.

## 4 Discussion

Lung cancer is a global health concern, representing the second most frequently diagnosed cancer and the leading cause of cancer-related deaths worldwide in 2020 (Jiang et al., 2021). Lung cancer often goes undiagnosed until it reaches an advanced stage, contributing to its high mortality rate. In recent years, the use of tissue and blood biomarkers has played a crucial role in guiding treatment decisions for advanced lung cancer patients (Wang et al., 2012; Morgensztern et al., 2015; Li et al., 2022). PARPi, which holds great promise as antitumor agents due to their ability to target PARP-1, a key factor contributing to tumor growth, increased malignancy, and the development of drug resistance (Malyuchenko et al., 2015). With the widespread approval and application of PARPi in various tumor types, this class of drugs has the potential to become the next “broad-spectrum anti-cancer miracle drug.”

In this study, we systematically analyzed twelve high-quality clinical trials involving 3,132 lung cancer patients, spanning ages from 27 to 89 years. Our primary focus was to evaluate the differences in PFS, OS, and AEs between treatment regimens containing PARPi and those without. Adhering to the 2020 draft guideline for cancer drug approval in clinical trials, we regarded PFS and OS as essential efficacy endpoints (Lee, 2018). Our results revealed that the incorporation of PARPi into lung cancer treatment regimens extended OS in patients with advanced lung cancer. Specifically, PARPi appeared to significantly benefit PFS in patients with small cell lung cancer. While individual experiments may occasionally yield contradictory conclusions, our comprehensive analysis provides a more accurate estimation of the effect size, resolves discrepancies among studies, and ultimately offers conclusive results when individual studies might be inconclusive. In the systematic review of OS, M. Catherine Pietanza et al.'s original study (Ramalingam et al., 2021) reported a positive effect of PARPi on lung cancer, improving OS. However, nine (Chabot et al., 2017; Ramalingam et al., 2017; Owonikoko et al., 2019; Garcia-Campelo et al., 2020; Ai et al., 2021; Argiris et al., 2021; Fennell et al., 2022; Govindan et al., 2022; Woll et al., 2022) other studies reported no significant difference between PARP inhibitor-containing regimens and non-PARP inhibitor regimens. Nevertheless, our meta-analysis results, based on data from ten studies, indicated a significant improvement in the OS of lung cancer patients with PARP inhibitor-containing regimens. Similarly, in the PFS analysis, two original studies (Owonikoko et al., 2019; Ai et al., 2021) suggested that PARP inhibitor-containing regimens could prolong the PFS of lung cancer patients. Yet, the combined analysis of all available experimental data indicated that PARP inhibitor-containing regimens did not extend PFS compared to non-PARP inhibitor regimens.

To account for differences in lung cancer types and treatment regimens, we conducted subgroup analyses. These revealed that in small cell lung cancer, treatment with PARPi alone or in combination with chemotherapy extended PFS. In non-small cell lung cancer, PARPi combined with chemotherapy prolonged OS. Additionally, when PARPi were combined with chemotherapy, both OS and PFS were extended in patients with LP52-positive non-small cell lung cancer. LP52 is a binary gene expression classifier based on the gene content of the expression-based Lung Subtype Panel (LSP) (Wilkerson et al., 2013; Faruki et al., 2016). But as far as the current study is concerned, LP52 was only used to predict adverse outcomes and improved responses to veliparib (Ramalingam et al., 2021; Govindan et al., 2022), not all PARPi. However, it is worth noting that the results of the two studies, Ramalingam et al. (2021) and Govindan et al. (2022), offered different conclusions regarding the impact of veliparib on lung cancer treatment. These disparities underscore the need for further investigation and highlight the potential influence of patient characteristics on treatment outcomes.

Several other studies, although not included in this meta-analysis due to the absence of a control group, reported some benefit. In the Phase 1/2 ATF-07 trial (NCT02412371), the PARP inhibitor veliparib (ABT-888), when combined with concurrent chemoradiotherapy, induced DNA damage in patients with stage III NSCLC. This approach yielded positive results, including an objective response rate (ORR) of 64.3% and a median progression-free survival (mPFS) of 19.6 months.

However, the Lung-MAP SWOG S1400G trial (NCT02154490) found that talazoparib (Talzenna) had a lower ORR of 4% in patients with advanced refractory lung squamous cell carcinoma, specifically in tumors with BRCA1/2, ATM, ATR, and PALB2 mutations (Owonikoko et al., 2021). The S1900A substudy of the LUNG-MAP trial evaluated the role of the PARP inhibitor rucaparib in advanced NSCLC with BRCA1/2 mutations or genomic loss of heterozygosity (LOH) as a phenotypic marker for homologous recombination deficiency (HRD). In this study, the ORR was 7%, and the disease control rate (DCR) was 62%. S1900A did not demonstrate the expected level of efficacy for rucaparib in patients with advanced NSCLC exhibiting high genomic LOH and/or BRCA1/2 mutations (Riess et al., 2021).

Combination of PARPi and Immune Checkpoint Inhibitors: The combination of PARPi with immune checkpoint inhibitors (ICI) presents a compelling and rational approach due to the well-established interplay between the DNA repair pathway and immune activation (Pilié et al., 2019). Preclinical studies in mouse models have demonstrated that PARPi can upregulate tumor PD-L1 expression and enhance tumor killing, surpassing the efficacy of either agent alone (Jiao et al., 2017). Recent reports have shown that co-administration of PARPi, such as niraparib, with anti-PD-1 agents can increase immune cell infiltration into the tumor microenvironment, leading to synergistic antitumor effects in various tumor types, including breast cancer, lung squamous cell carcinoma, colon adenocarcinoma, bladder cancer, and sarcoma (Wang et al., 2019). Similarly, combining the PARP inhibitor olaparib with PD-L1 blockade induced complete tumor regression in multiple immunocompetent SCLC mouse models (Sen et al., 2019). Notably, many tumor types that have been evaluated for the combination strategy have already demonstrated significant benefits from PARPi monotherapy but limited activity with ICIs. The next critical step is to identify the optimal patient populations that will derive the most benefit from this combination approach (Peyraud and Italiano, 2020).

The results of the meta-analysis revealed that PARP inhibitor-containing regimens were associated with a relatively higher incidence of hematologic toxicity but lower non-hematologic toxicity compared to non-PARP inhibitor regimens. Hematologic AEs, such as neutropenia, leukopenia, thrombocytopenia, and anemia, were more frequent with PARP inhibitor-containing regimens. However, hyponatremia was less common in the PARP inhibitor group. In terms of non-hematologic toxicity, PARP inhibitor-containing regimens were associated with lower rates of constipation, decreased appetite, diarrhea, and arthralgia but increased the risk of nausea, vomiting, and dyspnea. Importantly, no reports of deaths associated with PARPi were identified. In the context of advanced NSCLC, where multiple treatment options are available, maintaining patient quality of life and performance status (PS) is a crucial consideration (Hirsh, 2011; Hirsh et al., 2014). Moreover, our data did not identify any reports of deaths associated with PARPi. While this meta-analysis provides valuable insights into the potential of PARPi in lung cancer treatment, additional studies are necessary to further confirm their clinical utility.

The inclusion of PARPi in lung cancer treatment is a rapidly evolving field, and ongoing research is essential. One notable limitation is the heterogeneity in treatment regimens, including the use of PARPi alone or in combination with different therapies such as chemotherapy and radiotherapy. Future studies should consider potential sources of bias related to these variations. Additionally, the limited number of trials within some subgroups necessitates cautious interpretation, and results may evolve with the publication of new trials. Assessing publication bias was challenging due to the limited number of studies contributing to each outcome, and future research should address this issue. Furthermore, exploring the impact of patient characteristics, such as age and sex, as well as treatment cross-over between groups, is crucial in future studies. Moreover, the lack of effect sizes and confidence intervals for ORR results limited our ability to include them in the meta-analysis.

## 5 Conclusion

In conclusion, PARPi have emerged as a promising therapeutic option for advanced lung cancer. Our meta-analysis suggests that PARP inhibitor-containing regimens can improve OS, particularly in NSCLC and SCLC, while the impact on PFS varies by cancer type and treatment approach. The safety profile indicates an increased risk of specific hematological AEs, emphasizing the need for vigilant monitoring. Further research and clinical trials are essential to refine treatment strategies and identify patient populations that can benefit the most from PARP inhibitor therapy in lung cancer.

## Figures and Tables

**Table:** 

**PARP**	Poly (ADP-Ribose) Polymerase
**PARPi**	PARP inhibitors
**SCLC**	Small cell lung cancer
**NSCLC**	Non-small cell lung cancer
**LUAD**	Lung adenocarcinoma
**LUSC**	Lung squamous cell carcinoma
**OS**	Overall survival
**PFS**	Progression-free survival
**mOS**	Median overall survival
**mPFS**	Median progression-free survival
**ORR**	Objective response rate
**DCR**	Disease control rate
**PS**	Performance status
**AEs**	Adverse events
**LOH**	Loss of heterozygosity
**SSB**	single-strand breaks
**DSB**	double-strand breaks
**DDR**	DNA damage response
**HRD**	Homologous recombination deficiency
**HRR**	Homologous recombination repair
**BER**	base excision repair
**NER**	nucleotide excision repair
**MMR**	mismatch repair
**HR**	homologous recombination
**NHEJ**	non-homologous end joining
**TLS**	translesion synthesis
**ICL**	interstrand crosslink
**LSP**	Lung Subtype Panel
**RCT**	Randomized controlled trial
**HR**	Hazard ratio
**HRs**	Hazard ratios
**RRs**	Relative risks
**CI**	Confidence intervals
**MeSH**	Medical Subject Headings
**EMA**	European Medicines Agency
**FDA**	United States Food and Drug Administration
**PRISMA**	Preferred Reporting Items for Systematic Reviews and Meta-Analyses
**ECOG**	Eastern Cooperative Oncology Group
**ASCO**	American Society of Clinical Oncology
**ESMO**	European Society for Medical Oncology
**CTCAE**	Common Terminology Criteria for Adverse Events
**WBRT**	Whole-brain radiation therapy
**C**	Carboplatin/Cisplatin
**P**	Paclitaxel
**E**	Etoposide
**M**	Pemetrexed
**BID**	Twice a day
**NA**	Not available

## Data Availability

The original contributions presented in the study are included in the article/Supplementary Material, further inquiries can be directed to the corresponding authors.
