# Supplementary material for: Assessing the benefits and safety profile of incorporating poly ADP-ribose polymerase (PARP) inhibitors in the treatment of advanced lung cancer: a thorough systematic review and meta-analysis
Source: Front Pharmacol. 2024 Jun 21;15:1338442. doi: 10.3389/fphar.2024.1338442 (PMC11234112; doi:10.3389/fphar.2024.1338442)
Supplement: Supplementary file 1 [file Presentation1.pdf]

Retrieval strategy:

1.The statistical strategies on Pubmed

((((((((((olaparib) OR (AZD 2281)) OR (AZD221)) OR (Lynparza)) OR ((((rucaparib) OR (PF-01367338)) OR (Rubraca)) OR (AG 014699))) OR (((talazoparib) OR (Talzenna)) OR (BMN 673))) OR ((veliparib) OR (ABT 888))) OR (((niraparib) OR (Zejula)) OR (MK 4827))) OR ((PARP inhibitor) OR (poly ADP-ribose polymerase inhibitor))) AND (((Lung Neoplasm) OR (Lung Cancer)) OR (Pulmonary Cancer))) AND (Randomized controlled trial)

2.The statistical strategies on Cochrane

#1	(olaparib) OR (AZD 2281) OR (AZD221) OR (Lynparza)	S MeSH Limits	866
#2	(rucaparib) OR (PF-01367338) OR (Rubraca) OR (AG 014699)	Limits	176
#3	(talazoparib) OR (Talzenna) OR (BMN 673)	Limits	118
#4	(veliparib) OR (ABT 888)	Limits	261
#5	(niraparib) OR (Zejula) OR (MK 4827)	Limits	265
#6	(PARP inhibitor) OR (poly ADP-ribose polymerase inhibitor)	Limits	763
#7	(Lung Neoplasm) OR (Lung Cancer) OR (Pulmonary Cancer)	Limits	33794
#8	(Randomized controlled trial)	Limits	1008669
#9	#1 OR #2 OR #3 OR #4 OR #5 OR #6	Limits	1681
#10	#9 AND #7 AND #8	Limits	149

3.The statistical strategies on Embase

History Save | Delete | Print view | Export | Email Combine > using And Or Collapse

#10	#7 AND #8 AND #9	268
#9	#1 OR #2 OR #3 OR #4 OR #5 OR #6	28,793
#8	randomized AND controlled AND trial	1,110,629
#7	lung AND neoplasm OR (lung AND cancer) OR (pulmonary AND cancer)	743,823
#6	parp AND inhibitor OR (poly AND 'adp ribose' AND polymerase AND inhibitor)	21,453
#5	niraparib OR zejula OR (mk AND 4827)	2,739
#4	veliparib OR (abt AND 888)	2,970
#3	talazoparib OR talzenna OR (bmn AND 673)	2,119
#2	rucaparib OR 'pf 01367338' OR rubraca OR (ag AND 014699)	2,666
#1	'olaparib'/exp OR olaparib OR (azd AND 2281) OR azd221 OR 'lynparza'/exp OR lynparza	9,971
